# **PlMYB3RL homodimer activates *PlSGRL* to accelerate high temperature**–**induced leaf senescence by promoting chlorophyll degradation in herbaceous peony (*Paeonia lactiflora* Pall.)**

**DOI:** 10.1093/hr/uhag135

**Published:** 2026-04-13

**Authors:** Mengting Zu, Jiajia Zuo, Daqiu Zhao, Jun Tao

**Affiliations:** College of Horticulture and Landscape Architecture, Yangzhou University, Yangzhou 225009, China; College of Horticulture and Landscape Architecture, Yangzhou University, Yangzhou 225009, China; College of Horticulture and Landscape Architecture, Yangzhou University, Yangzhou 225009, China; College of Horticulture and Landscape Architecture, Yangzhou University, Yangzhou 225009, China

## Abstract

High-temperature stress caused by global warming can induce leaf senescence, which adversely affects plant growth and agricultural productivity worldwide. However, the molecular mechanism of high temperature–induced leaf senescence remains largely unexplored. In this study, a Stay-green-like protein (PlSGRL) that regulates chlorophyll degradation was identified in herbaceous peony (*Paeonia lactiflora* Pall.). PlSGRL was localized to the chloroplasts, and its expression was upregulated under high-temperature stress. Virus-induced *PlSGRL* silencing markedly delayed high temperature–induced leaf senescence in *P. lactiflora*, as evidenced by higher chlorophyll content, sustained photosystem II efficiency, and alleviated oxidative damage—reflected in reduced malondialdehyde content, relative electrical conductivity, and reactive oxygen species accumulation. Conversely, *PlSGRL* overexpression accelerated high temperature–induced leaf senescence. Subsequently, we identified an atypical MYB transcription factor, PlMYB3RL, that directly promoted *PlSGRL* expression. PlMYB3RL was localized to the nucleus, and its expression was also upregulated under high-temperature stress. Similarly, *PlMYB3RL* silencing significantly delayed high temperature–induced leaf senescence. Additionally, PlMYB3RL formed a homodimer through self-interaction, and this homodimer enhanced the transcriptional activation of *PlSGRL* in a dose-dependent manner. Collectively, these data demonstrate that the PlMYB3RL homodimer activates *PlSGRL* expression to promote chlorophyll degradation and leaf senescence under high-temperature stress in *P. lactiflora*. These findings reveal a novel regulatory module underlying high temperature–induced leaf senescence in *P. lactiflora*, providing key gene resources and theoretical support for breeding high-temperature-resistant cultivars.

## Introduction

As global climates shift, extreme weather events are becoming more frequent and severe, with high-temperature stress posing a serious risk to crop production and ecosystem functioning [1, 2]. High-temperature stress severely impairs seed germination, hampers plant growth and development, and triggers earlier leaf senescence [3–5]. By 2100, global temperatures are expected to rise by 1.8 to 4.0°C [6]. Notably, in major wheat-growing regions, a temperature increase of 2°C during the growing season accelerated leaf senescence and reduced grain yield by as much as 50% [7]. Moreover, high-temperature stress induced earlier leaf senescence in soybeans, resulting in significant reductions in seed fullness and seed size, with the final yield decreasing by nearly 30% [3]. Collectively, high temperature–induced leaf senescence substantially compromises the economic viability of agricultural crops. However, the molecular mechanisms of this phenomenon remain largely unexplored.

Leaf senescence is a fine-tuned genetically programmed process that is traditionally regarded as a developmental transition [8, 9]. However, abiotic stresses, including high temperature, drought, and darkness, can disrupt this process and trigger premature leaf senescence [10–12]. High-temperature stress has been shown to disrupt reactive oxygen species (ROS) homeostasis, impair photosystem II efficiency, and accelerate chlorophyll loss, ultimately leading to premature senescence [13–15]. Among these physiological changes, chlorophyll loss is a hallmark event of leaf senescence. Increasing evidence indicates that chlorophyll loss during high temperature–induced leaf senescence is primarily due to accelerated chlorophyll degradation rather than suppressed chlorophyll biosynthesis [16, 17]. Chlorophyll degradation is mediated by the coordinated action of chlorophyll catabolic enzymes (CCEs) and their regulatory factors [18]. Genetic studies have demonstrated that disruption of key CCEs, such as PPH and NOL, can significantly delay high temperature–induced leaf senescence and maintain photosynthetic performance, as evidenced by improved chlorophyll retention, photochemical efficiency, and membrane stability [16, 19]. Stay-green (SGR) proteins, along with CCEs, contribute significantly to the initiation of chlorophyll degradation by disturbing pigment-protein complexes within chloroplasts [20, 21]. In *Arabidopsis thaliana*, the SGR family consists of AtSGR1, AtSGR2, and AtSGRL, which exhibit distinct yet coordinated roles in chlorophyll catabolism [22–25]. Notably, *AtSGRL* overexpression accelerated chlorophyll degradation and chlorophyll-protein complex disassembly synergistically with AtSGR1 through homo−/heterodimer formation and interactions with AtLHCII and CCEs under salt stress [26]. However, its exact function in high temperature–induced chlorophyll degradation is still poorly understood.

To date, multiple transcription factor families, such as MYB, NAC, and WRKY, have been shown to bind to the *SGR* promoters in different plant species, thereby fine-tuning chlorophyll degradation in response to environmental stresses [27–29]. These studies indicate that *SGR*-mediated chlorophyll degradation is controlled by a complex transcriptional regulatory network rather than by a single class of upstream regulators. Among these transcription factors, the MYB family is one of the largest and most functionally diverse transcription factor families in plants and has been widely implicated in the regulation of chlorophyll degradation and leaf senescence. In kiwifruit, AdMYB7 directly activated *AdSGR* transcription and promoted chlorophyll degradation during ripening [30]. In rice, MYB-related RADIALIS-LIKE3 (RL3) enhanced *OsSGR* expression during the dark-induced senescence process [31], whereas OsMYB102 negatively regulated senescence by suppressing *OsSGR* [32]. Collectively, these findings demonstrate that typical MYB transcription factors can finely tune *SGR* expression in various biological contexts. However, despite the well-established roles of typical MYB proteins, to date, no studies have reported on the role of atypical MYB transcription factors in regulating high temperature–induced leaf senescence.

Herbaceous peony (*Paeonia lactiflora* Pall.) is a major ornamental crop, renowned not only for its vibrant blooms and cut-flower economic value, but also for its medicinal properties [33]. In temperate regions, summer high temperatures caused by global warming frequently induce premature leaf senescence with chlorophyll degradation in *P. lactiflora*. This process severely impairs plant vigor and nutrient accumulation, thereby significantly impacting the development of the herbaceous peony industry [34, 35]. Therefore, elucidating the molecular mechanisms underlying high temperature–induced chlorophyll degradation in *P. lactiflora* is of great significance. In our previous study [35], transcriptome sequencing analysis revealed that *PlSGRL* is one of the key candidate genes involved in chlorophyll degradation and leaf senescence under high-temperature stress. Therefore, this study aimed to elucidate the molecular mechanisms of high temperature–induced leaf senescence in *P. lactiflora* by functionally characterizing *PlSGRL* (a homolog of the *SGR* gene family) and identifying its upstream regulator, PlMYB3RL. To achieve this, we employed phylogenetic analysis, gene silencing/overexpression, promoter screening, and protein interaction assays. These investigations advance our understanding of both high temperature–induced leaf senescence mechanisms and the regulatory roles of atypical MYB transcription factors in abiotic stress responses.

## Results

### Phylogenetic and functional characterization of *PlSGRL*

**Figure 1:**
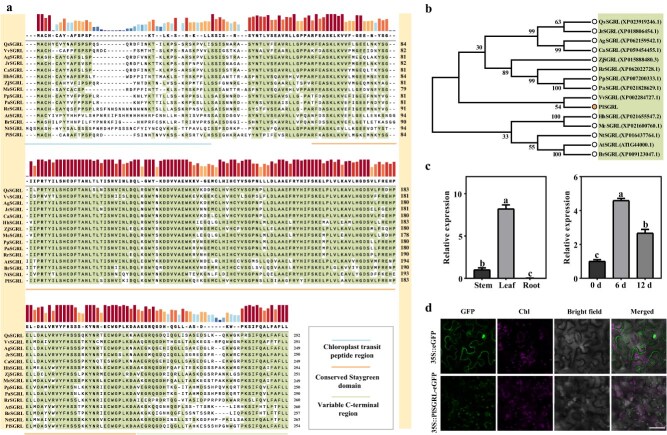
Phylogenetic and expression analysis of *PlSGRL*. (a) Multiple sequence alignment of PlSGRL and its homologs. The conserved chloroplast transit peptide region is highlighted in blue, the conserved Staygreen domain in orange, and the variable C-terminal region in green. (b) Phylogenetic tree of PlSGRL and other homologs. The neighbor-joining consensus tree was constructed in MEGA 7.0 using *p*-distance evolutionary distances with 1000 bootstrap replicates, support values are indicated at nodes. (c) Relative *PlSGRL* expression in the leaf, stem, and root of *P. lactiflora* at designated time points under high-temperature stress. (d) Subcellular localization of PlSGRL. Chl, chloroplast autofluorescence. Bar = 50 μm. Data are presented as mean ± SD (*n* = 3). Different letters indicate significant differences (*p* < 0.05).

In our previous study, a Stay-green-like protein (isoform 113183) was identified as a candidate gene positively regulating high temperature–induced leaf senescence in *P. lactiflora* by accelerating chlorophyll degradation [35]. In this study, we cloned its full-length cDNA via PCR, which contains a 765-bp coding sequence (CDS) that encodes a protein of 254 amino acids. To assess its evolutionary relationship with other homologs, we downloaded fourteen SGRL proteins from the NCBI database. Multiple sequence alignment revealed that isoform 113183 shares high homology with the SGRL family in other plant species and contained a conserved Staygreen domain critical for chlorophyll degradation. Furthermore, these proteins all possess a conserved N-terminal chloroplast transit peptide (TP) and a variable C-terminal region (Fig. 1a). Phylogenetic analysis showed that isoform 113183 clustered with VvSGRL, sharing 71% amino acid sequence identity (Fig. 1b). These results indicate that isoform 113183 belongs to the SGRL family and was therefore named PlSGRL. Expression analysis showed that *PlSGRL* is expressed at higher levels in leaves than in stems and roots. Under high-temperature stress, *PlSGRL* expression initially increased and then decreased, reaching 4.59 times the Day 0 level on Day 6 and 2.66 times on Day 12 (Fig. 1c). These results indicated that PlSGRL is evolutionarily conserved and responsive to high-temperature stress.

Next, to assess its subcellular localization, PlSGRL was fused to eGFP and expressed in *Nicotiana benthamiana* leaves. Fluorescence signals from the 35S::PlSGRL-eGFP fusion protein colocalized with chloroplast autofluorescence, confirming that PlSGRL localizes to the chloroplasts, which reinforced its suggested involvement in chlorophyll degradation (Fig. 1d).

### *PlSGRL* silencing delays high temperature–induced leaf senescence in *P. lactiflora*

**Figure 2:**
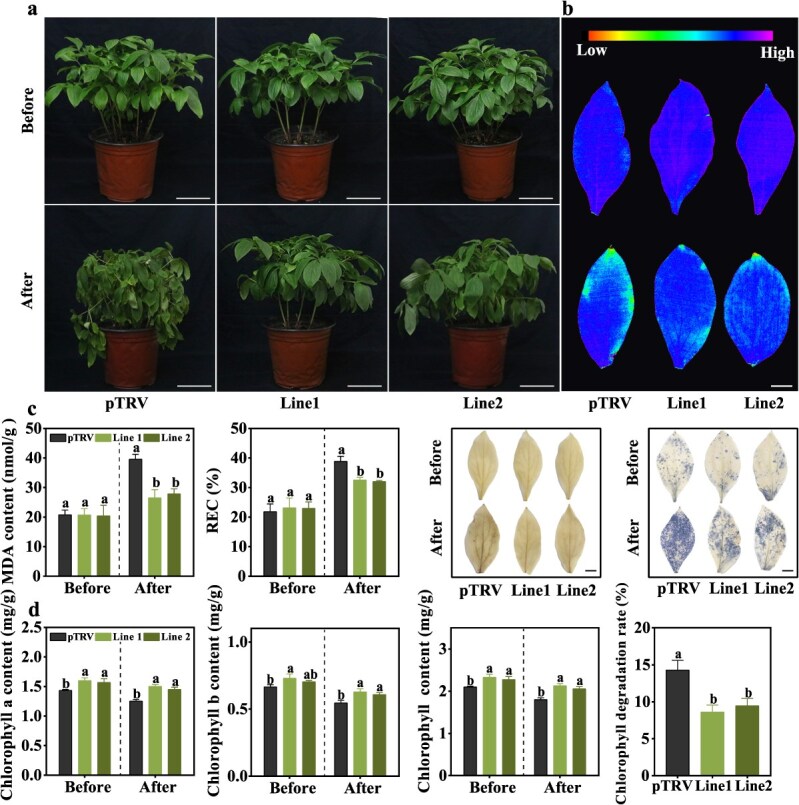
Effect of high-temperature stress on the pTRV and *PlSGRL*-silenced *P. lactiflora* plants. (a) Phenotype of the pTRV and *PlSGRL*-silenced plants after high-temperature stress. Bars = 10 cm. (b) Maximum photochemical efficiency of photosystem II (F_v_/F_m_) value of the pTRV and *PlSGRL*-silenced plants. Bar = 1 cm. (c) Malondialdehyde (MDA) content, REC, and ROS accumulation (DAB and NBT staining) in the pTRV and *PlSGRL*-silenced plants. Bars = 1 cm. (d) Chlorophyll a, b content, total chlorophyll, and degradation rates in the pTRV and *PlSGRL*-silenced plants. Data are presented as mean ± SD (*n* = 3). Different letters indicate significant differences (*P* < 0.05).

*PlSGRL* expression increased under high-temperature stress, implying that it might play a critical role in high temperature–induced leaf senescence. To confirm this assumption, virus-induced gene silencing (VIGS) was employed in *P. lactiflora*. The pTRV control (pTRV1 and empty pTRV2 vector) and two independent *PlSGRL*-silenced lines were used in this study. The transcript levels of *PlSGRL* were reduced by 54% and 50% in these two silenced lines, respectively, compared with the pTRV control (Supplementary Fig. S1). These plants were exposed to high-temperature stress and evaluated for phenotypic changes. Figure 2a demonstrates that the *PlSGRL*-silenced plants maintained greener leaves and exhibited a significantly delayed high temperature–induced leaf senescence phenotype, supported by decreased a* (red-green axis), reduced b* (yellow-blue axis), and increased *h*° (hue angle) under high-temperature stress (Supplementary Fig. S2). Moreover, these *PlSGRL*-silenced plants maintained higher photosystem II efficiency, as evidenced by elevated maximum photochemical efficiency of photosystem II (Fv/Fm) values (Fig. 2b). Simultaneously, they showed a marked reduction in oxidative damage, with lower malondialdehyde (MDA) content, lower relative electrical conductivity (REC) levels, and diminished ROS accumulation as observed through diaminobenzidine (DAB) and nitroblue tetrazolium (NBT) staining (Fig. 2c). Notably, the *PlSGRL*-silenced plants retained higher chlorophyll content under high-temperature stress. The chlorophyll degradation rates in the *PlSGRL*-silenced plants were 8% and 9%, significantly lower than the rate of 14% observed in the pTRV control plants (Fig. 2d). These results underscored the necessity of *PlSGRL* in accelerating high temperature–induced leaf senescence.

### *PlSGRL* overexpression accelerates high temperature–induced leaf senescence in *Nicotiana tabacum*

**Figure 3:**
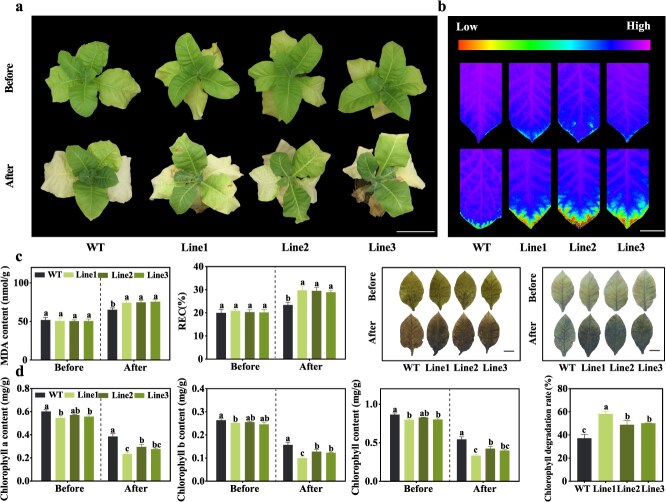
Effect of high-temperature stress on the WT and *PlSGRL*-overexpressing *N. tabacum* plants. (a) Phenotype of WT and *PlSGRL*-overexpressing plants after high-temperature stress. Bar = 10 cm. (b) F_v_/F_m_ values of the WT and *PlSGRL*-overexpressing plants. Bar = 5 cm. (c) MDA content, REC levels, and ROS accumulation (DAB and NBT staining) in the WT and *PlSGRL*-overexpressing plants. Bars = 5 cm. (d) Chlorophyll a, b content, total chlorophyll, and degradation rates in the WT and *PlSGRL*-overexpressing plants. Data are presented as mean ± SD (*n* = 3). Different letters indicate significant differences (*P* < 0.05).

To further investigate the function of *PlSGRL* in accelerating high temperature–induced leaf senescence, transgenic *Nicotiana tabacum* plants overexpressing *PlSGRL* were generated, and positive transgenic lines were confirmed by molecular identification (Supplementary Fig. S3). These plants were then subjected to high-temperature stress. After 12 days of high-temperature stress, the *PlSGRL*-overexpressing plants displayed accelerated leaf yellowing and high temperature–induced senescence, as indicated by increased a*, elevated b*, and decreased *h*° under high-temperature stress (Fig. 3a and Supplementary Fig. S4). In line with the observed phenotypic differences, the *PlSGRL*-overexpressing plants showed lower F_v_/F_m_ values (Fig. 3b), accompanied by elevated MDA content, increased REC levels, and enhanced ROS accumulation (Fig. 3c). Notably, the chlorophyll content in the *PlSGRL*-overexpressing plants decreased more rapidly than in the wild-type (WT) plants. After high-temperature stress, the chlorophyll degradation rates of three independent overexpressing lines reached 58%, 49%, and 50%, which were significantly higher than the 37% in the WT plants (Fig. 3d). These results further confirm the conserved function of *PlSGRL* in accelerating high temperature–induced leaf senescence by promoting chlorophyll degradation.

### PlMYB3RL is identified as a putative regulator of *PlSGRL*

**Figure 4:**
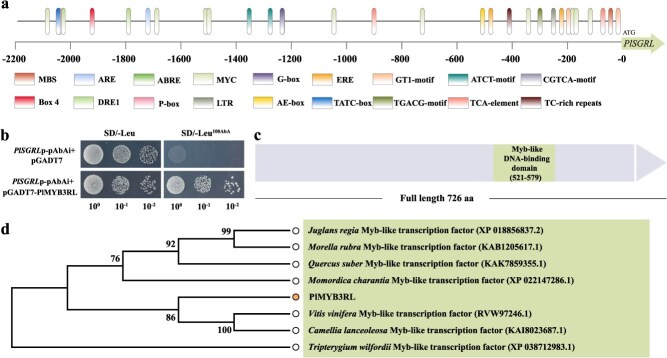
Evidence for PlMYB3RL-mediated regulation of the *PlSGRL* promoter and phylogenetic analysis of PlMYB3RL. (a) Cis-acting elements of the *PlSGRL* promoter. (b) Y1H assay to investigate the interaction between the *PlSGRL* promoter and PlMYB3RL. The yeast bait cells (transformed with *PlSGRL* promoter) harboring pGADT7-PlMYB3RL prey plasmids and pGADT7 empty vector were selected on SD/-Leu medium with 100 ng ml^−1^ AbA for interaction detection. AbA, aureobasidin A. (c) Schematic of PlMYB3RL protein. (d) Phylogenetic tree analysis comprising PlMYB3RL with other homologs.

To identify upstream regulatory factors of *PlSGRL* during high temperature–induced leaf senescence, we cloned the promoter region of *PlSGRL*. Its 2218-bp promoter sequence contains a diverse set of cis-acting elements, including MYB-binding sites (MBS), abscisic acid-responsive elements (ABRE), MYC-binding motifs, and other elements associated with stress responses (e.g., DRE1, LTR) and hormone signaling (e.g., P-box, TCA-element) (Fig. 4a). Subsequently, a yeast one-hybrid (Y1H) screening using the *PlSGRL* promoter as bait was performed against a *P. lactiflora* cDNA library, and a MYB transcription factor caught our attention (Fig. 4b). It consists of a 2181-bp CDS encoding 726 amino acids and contains a conserved Myb DNA-binding domain (Fig. 4c). BLASTP analysis against the TAIR database indicate that it is homologous to At5g41020 from *A. thaliana* (Supplementary Fig. S5), encoding an atypical AtMYB3R-like transcription factor [36]. Thus, it was named PlMYB3RL. Given that the function of this atypical AtMYB3R-like transcription factor has not yet been studied in plants, we focused on it for further investigation. Phylogenetic analysis showed that PlMYB3RL was clustered with *Vitis vinifera* Myb-like transcription factor (RVW97246.1) and *Camellia lanceoleosa* Myb-like transcription factor (KAI8023687.1) (Fig. 4d). Collectively, these findings suggested that PlMYB3RL might act as an atypical MYB transcription factor involved in the transcriptional regulation of *PlSGRL*.

### PlMYB3RL functions as a nuclear-localized transcriptional activator

**Figure 5:**
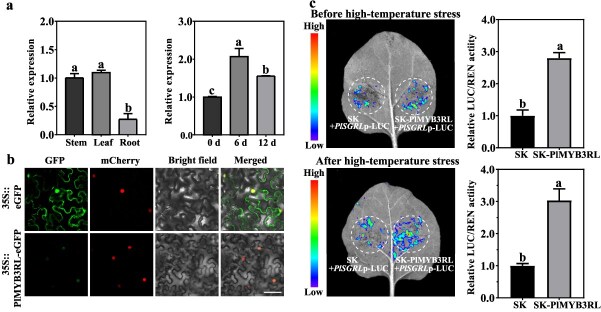
Expression characteristics of PlMYB3RL and the evidence for its activation of the *PlSGRL* promoter. (a) *PlMYB3RL* expression in *P. lactiflora* leaf, stem, and root at designated time points under high-temperature stress. (b) Subcellular localization of PlMYB3RL in *N. benthamiana* leaves. The mCherry protein, which marks the nucleus, was co-infiltrated with 35S::eGFP or 35S::PlMYB3RL-eGFP fusion vectors. Bar = 50 μm. (c) DLR assay between *PlSGRL* promoter and PlMYB3RL. The LUC/REN ratio of SK + *PlSGRLp*-LUC was normalized to 1. LUC activity was also imaged after the application of 1 mM D-luciferin potassium salt. Data are presented as mean ± SD (*n* = 3). Different letters indicate significant differences (*P* < 0.05).

Since the functions of PlMYB3RL and its homologs have not been fully characterized, we first assessed the *PlMYB3RL* expression levels across different tissues. The results indicated that *PlMYB3RL* was more highly expressed in leaves and stems than in roots. Under high-temperature stress, *PlMYB3RL* expression initially increased and then decreased, reaching 2.07-fold that of Day 0 on Day 6 and 1.56-fold that of Day 0 on Day 12. This expression pattern was correlated with that of *PlSGRL*, indicating a potential regulatory relationship between the two (Fig. 5a). Subcellular localization analysis showed that the control eGFP was distributed throughout the cell, whereas the PlMYB3RL-eGFP fusion protein and the nuclear localization signal marker were colocalized, indicating that PlMYB3RL localizes to the nucleus (Fig. 5b).

To assess the regulatory effect of PlMYB3RL on the *PlSGRL* promoter, we conducted a dual-luciferase reporter (DLR) assay. Co-expression of PlMYB3RL and the *PlSGRL* promoter-LUC reporter significantly increased luciferase activity compared to empty vector controls (Fig. 5c). These results demonstrate that PlMYB3RL functions as a nuclear-localized transcription factor that activates the transcription of *PlSGRL* under high-temperature stress.

### *PlMYB3RL* silencing delays high temperature–induced leaf senescence in *P. lactiflora*

**Figure 6:**
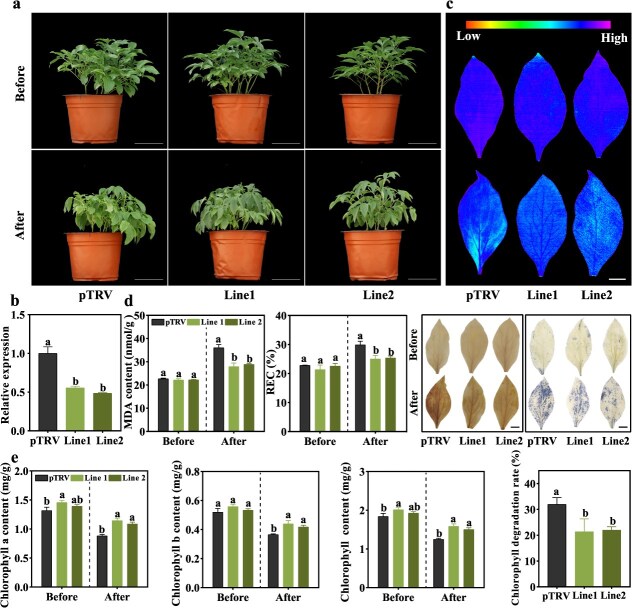
Effects of high-temperature stress on the pTRV and *PlMYB3RL*-silenced *P. lactiflora* plants. (a) Phenotype of the pTRV and *PlMYB3RL*-silenced plants after high-temperature stress. Bars = 10 cm. (b) Relative expression of *PlSGRL* in the *PlMYB3RL*-silenced plants based on VIGS. (c) F_v_/F_m_ values of the pTRV and *PlMYB3RL*-silenced plants. Bar = 1 cm. (d) MDA content, REC levels, and ROS accumulation (DAB and NBT staining) in the pTRV and *PlMYB3RL*-silenced plants. Bars = 1 cm. (e) Chlorophyll a, b content, total chlorophyll, and degradation rates in the pTRV and *PlMYB3RL*-silenced plants. Data are presented as mean ± SD (*n* = 3). Different letters indicate significant differences (*P* < 0.05).

To clarify the function of *PlMYB3RL* in high temperature–induced leaf senescence, the pTRV control and two independent *PlMYB3RL*-silenced lines were used. The transcript levels of *PlMYB3RL* were reduced by 37% and 52% in these two silenced lines, respectively, compared with the pTRV control (Supplementary Fig. S6). After high-temperature treatment, the *PlMYB3RL*-silenced plants exhibited a dramatically delayed high temperature–induced leaf senescence phenotype (Fig. 6a) supported by decreased a*, reduced b*, and increased *h*°, with a concurrent significant downregulation of *PlSGRL* expression (Supplementary Fig. S7, Fig. 6b), further indicating a regulatory relationship between PlMYB3RL and *PlSGRL*. Corresponding to the observed phenotypic differences, the *PlMYB3RL*-silenced plants maintained higher Fv/Fm values and exhibited alleviated oxidative damage, as evidenced by the reduction in MDA content, REC levels, and ROS accumulation (Fig. 6c, d). Particularly, the *PlMYB3RL*-silenced plants retained higher chlorophyll content. After high-temperature stress, the chlorophyll degradation rates in the *PlMYB3RL*-silenced plants were 21% and 22%, which were significantly lower than the 32% observed in the pTRV plants (Fig. 6e). All these results were consistent with those observed in the *PlSGRL*-silenced plants, suggesting that PlMYB3RL positively regulates the transcriptional activation of *PlSGRL* during high-temperature stress.

### Self-interaction of PlMYB3RL enhances its transcriptional activation of *PlSGRL*

**Figure 7:**
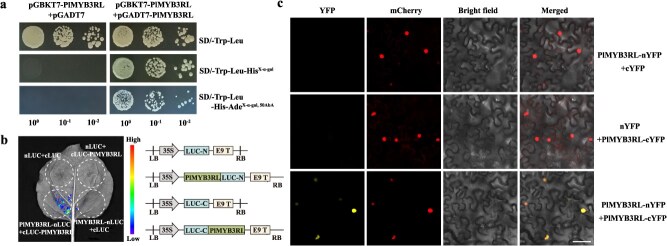
PlMYB3RL physically interacts with itself. (a) Y2H assay. The yeast cells transformed with prey (pGADT7-PlMYB3RL) and bait (pGBKT7-PlMYB3RL), along with negative control, were selected on SD/-Trp-Leu-His-Ade + X-α-gal medium with 50 ng ml^−1^ AbA for interaction detection. AbA, aureobasidin A. (b) LCA. (c) BiFC assay. PlMYB3RL was fused into the nYFP and cYFP vectors, respectively. The indicated combinations of nYFP and cYFP vectors, along with an mCherry protein containing a nuclear localization signal, were co-infiltrated into *N. benthamiana* leaves. Bars = 50 μm.

Yeast two-hybrid (Y2H) screening was employed to identify potential interactors of PlMYB3RL, and surprisingly, PlMYB3RL itself was identified among the positive clones. Yeast cells cotransfected with pGBKT7-PlMYB3RL and pGADT7-PlMYB3RL, exhibited normal growth and turned blue when cultured on SD/-Trp-Leu-His-Ade medium supplemented with X-α-gal and 50 ng ml^−1^ AbA (Fig. 7a). Subsequently, a luciferase complementation assay (LCA) was performed. It was found that a strong luminescence signal was detected only in *N. benthamiana* leaves co-infiltrated with PlMYB3RL-nLUC and PlMYB3RL-cLUC (Fig. 7b). Furthermore, a bimolecular fluorescence complementation (BiFC) assay was carried out in *N. benthamiana* leaves. The yellow fluorescent protein (YFP) signal was detected in the nucleus when PlMYB3RL-nYFP and PlMYB3RL-cYFP were co-expressed (Fig. 7c). These results confirm that PlMYB3RL interacted with itself to form the homodimer in vivo.

**Figure 8:**
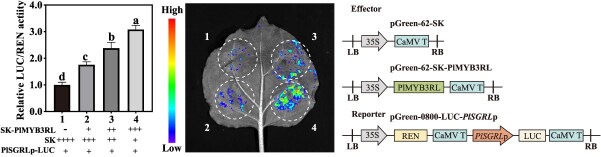
DLR assay evaluating the effect of PlMYB3RL homodimerization on its transactivation of the *PlSGRL* promoter. Increasing amounts of the PlMYB3RL effector were co-expressed with a constant amount of the *PlSGRL*p-LUC reporter. Data are presented as mean ± SD (n = 3). Different letters indicate significant differences (*P* < 0.05).

Subsequently, a DLR assay was conducted to assess the effect of the PlMYB3RL homodimer on its transactivation of *PlSGRL*. The results revealed that with increasing amounts of the PlMYB3RL effector, the luciferase activity driven by the *PlSGRL* promoter increased in a dose-dependent manner (Fig. 8). This suggests that PlMYB3RL homodimerization enhances its capacity to activate *PlSGRL* expression under high-temperature stress.

## Discussion

**Figure 9:**
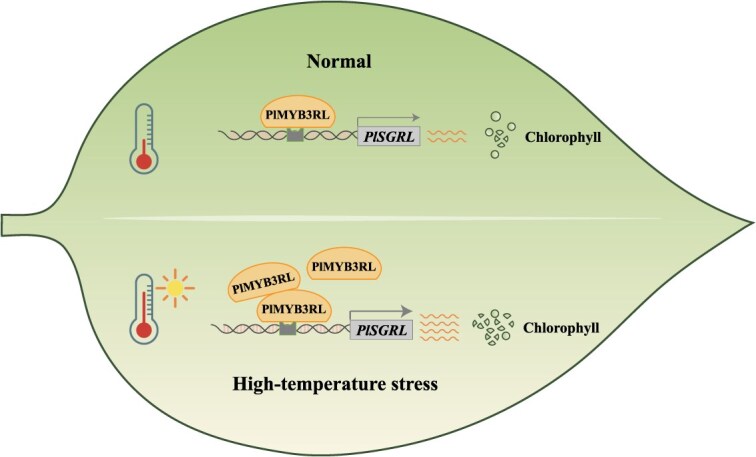
A proposed PlMYB3RL-*PlSGRL* regulatory model under high-temperature stress in *P. lactiflora*. Under normal conditions, PlMYB3RL is expressed at a basal level, which only weakly promotes the transcription of PlSGRL. Consequently, the expression of PlSGRL remains relatively low, and the rate of chlorophyll degradation is maintained at a low level. In contrast, under high-temperature stress, the expression of PlMYB3RL is upregulated and forms homodimers, which significantly enhances the transcriptional activation of *PlSGRL*. As a result, the elevated *PlSGRL* expression accelerates the rate of chlorophyll degradation.

Under global climate change, the molecular mechanisms underlying high temperature–induced leaf senescence remain poorly understood. As a perennial herb, *P. lactiflora* represents an ideal model system for investigating this process. This is largely due to its high susceptibility to high-temperature stress, which readily triggers premature leaf senescence and subsequently compromises plant development. This study elucidated a novel transcriptional regulatory module in which the transcription factor PlMYB3RL activates its target gene *PlSGRL*, thereby accelerating high temperature–induced leaf senescence in *P. lactiflora* by promoting chlorophyll degradation (Fig. 9).

### *PlSGRL* accelerates high temperature–induced leaf senescence through promoting chlorophyll degradation

The SGR family plays a pivotal role in regulating chlorophyll degradation [20]. Phylogenetic analysis revealed that PlSGRL exhibited high homology with previously characterized SGRL proteins in other plants, sharing a conserved Staygreen domain critical for promoting chlorophyll degradation by binding to chlorophyll-protein complexes [37]. Moreover, similar to other homologs, PlSGRL localized in the chloroplast, which was consistent with its function in chlorophyll degradation [38]. Although SGRL is expressed in presenescent tissues and participates in chlorophyll turnover [39], it primarily functions in stress-induced chlorophyll degradation. In *A. thaliana*, AtSGRL interacts with AtSGR1 and CCEs at thylakoid membrane complexes to promote chlorophyll degradation under high-salinity, osmotic, and drought stresses [26, 40]. In *Oryza sativa*, *OsSGRL* expression decreased during natural and dark-induced leaf senescence, while its overexpression promoted chlorophyll degradation [41]. In *Pisum sativum*, PsSGRL regulated chlorophyll degradation by facilitating the turnover of chlorophyll from damaged photosystems [42]. However, the role of SGRL under high-temperature stress has not yet been investigated.

Chloroplasts, being the sites of photosynthesis, are prime targets of high-temperature stress [43]. High-temperature stress disrupts thylakoid membrane integrity, impairs photosynthetic function, induces ROS accumulation, and promotes chlorophyll degradation [44]. Under high-temperature stress, cucumber CsABI5 promoted chlorophyll degradation by directly activating the pheophytinase (*CsPPH*) gene and the pheophorbide A oxygenase (*CsPAO*) gene, while countering CsMYB44’s suppression of these genes through physical interaction and the ubiquitin-mediated degradation of CsMYB44 [45]. In this study, high-temperature stress significantly induced *PlSGRL* expression, aligning with transcriptomic data from field samples during summer high temperatures [35], indicating its critical role in high temperature–induced chlorophyll degradation in *P. lactiflora*. Notably, in the later stages of high-temperature stress, this inducing effect diminished, suggesting that *P. lactiflora* might maintain a balance between protecting against high-temperature stress and the costs of survival by precisely regulating the expression level of *SGRL*. We further demonstrated that *PlSGRL* silencing significantly delayed leaf senescence and chlorophyll degradation in *P. lactiflora*, whereas its overexpression in *N. tabacum* accelerated these processes. These results indicate that *PlSGRL* plays an essential role in accelerating high temperature–induced leaf senescence by promoting chlorophyll degradation.

### PlMYB3RL activates *PlSGRL* to accelerate high temperature–induced leaf senescence

Although numerous studies have demonstrated the involvement of transcription factors in *SGR* regulation, the upstream transcriptional regulation of *SGRL* remains poorly characterized. In *Solanum lycopersicum*, *SlSGRL* expression is transcriptionally regulated by SlABI5 and SlABI5-like, which function downstream of ABA signaling to promote chlorophyll degradation under abiotic stress [46]. These transcription factors directly modulate *SlSGRL* transcription under abiotic stress conditions, thereby linking stress-responsive hormonal signaling to chlorophyll degradation. However, the transcriptional regulatory mechanisms governing *SGRL* under high-temperature stress remain elusive. Through Y1H screening, we identified PlMYB3RL as a transcriptional activator of *PlSGRL* under high-temperature stress. Phylogenetic analysis revealed that PlMYB3RL clustered with *A. thaliana* At5g41020, an atypical MYB transcription factor [36]. In this study, high-temperature stress induced the expression of *PlMYB3RL*, which was similar to the expression pattern of *PlSGRL*. Nuclear localization analysis showed that PlMYB3RL was a nuclear-localized protein, and the DLR assay demonstrated that PlMYB3RL could significantly increase the luciferase activity driven by the *PlSGRL* promoter, thus confirming that PlMYB3RL acted as a transcriptional activator of *PlSGRL*. Notably, the *PlMYB3RL-*silenced plants exhibited similar phenotypes to those of the *PlSGRL-*silenced plants, showing delayed leaf senescence and chlorophyll degradation.

### PlMYB3RL homodimer enhances its transcriptional regulation of *PlSGRL*

Transcription factor dimerization is a prevalent strategy for enhancing DNA-binding affinity and recruiting coactivators [47, 48]. In plants, dimerization is widely reported to enhance the stability and DNA-binding ability of MYB proteins, thereby improving their transcriptional regulatory efficiency under various environmental stresses. For instance, the IbMYB73 homodimer represses ABA signaling by directly activating *IbGER5*, thereby enhancing abiotic stress tolerance [49], while BplMYB46 forms homodimers and heterodimers to strengthen binding to MYBCORE motifs and improve salt and osmotic stress tolerance [50]. Similarly, GhMYB1a homodimerization enables coordinated regulation of flavonol and anthocyanin biosynthesis through transcriptional activation of key metabolic genes [51].

Beyond increasing transcriptional efficiency, homodimerization may provide an important regulatory advantage by conferring dose-dependent control over downstream gene activation. In this study, PlMYB3RL was shown to form a homodimer in vivo, and increasing PlMYB3RL abundance progressively enhanced *PlSGRL* promoter activity. Such a dose-dependent, homodimer-mediated activation mechanism may allow plants to finely tune the onset and progression of leaf senescence in response to high-temperature stress. Under mild high-temperature stress, limited PlMYB3RL accumulation may result in weak homodimer formation and restrained *PlSGRL* activation, thereby preventing premature chlorophyll degradation. In contrast, sustained or severe high-temperature stress could promote higher PlMYB3RL levels, favor homodimer assembly, and robustly activate *PlSGRL*, accelerating chlorophyll degradation and senescence. This regulatory mode is likely particularly advantageous for perennial species such as *P. lactiflora*, in which inappropriate or excessive senescence could compromise long-term growth and reproductive capacity. By coupling *PlSGRL* activation to both PlMYB3RL abundance and homodimer formation, plants may achieve a balanced trade-off between stress adaptation and survival costs, ensuring that senescence is initiated only when high-temperature stress exceeds a critical threshold. Notably, similar dose-dependent regulatory features have been observed in our previous study, where increasing levels of PlWRKY47–PlWRKY47 homodimers or PlWRKY72–PlWRKY47 heterodimers progressively enhanced *PlGAPC2* transcription, which in turn improved high-temperature tolerance by suppressing ROS accumulation in *P. lactiflora* [52]. These results underscore the potential universality of dose-dependent transcription factor dimerization as a strategy to fine-tune stress-responsive gene expression in *P. lactiflora* under high-temperature conditions.

## Conclusion

Through integrated molecular and physiological analyses, this study clarified the role of *PlSGRL* in accelerating high temperature–induced leaf senescence through promoting chlorophyll degradation in *P. lactiflora.* Moreover, we established a transcriptional regulatory network, wherein PlMYB3RL homodimer activated *PlSGRL* expression to accelerate leaf senescence. These findings expand the functional diversity of the SGR family and unveil a novel role for atypical MYB homodimers in high-temperature stress responses. This study offers a theoretical basis for breeding high temperature–resistant cultivars and potential targets for alleviating high temperature–induced leaf senescence in *P. lactiflora*.

## Materials and methods

### Plant materials and treatments

*Paeonia lactiflora* cultivar ‘Da Fugui’ plants were grown in the greenhouse at the College of Horticulture and Landscape Architecture, Yangzhou University. When the plants reached full maturity, their roots, stems, and leaves were harvested for tissue-specific analysis of *PlSGRL* and *PlMYB3RL* expression. In addition, three individual plants were exposed to high-temperature stress in a growth chamber. The chamber was set to a day/night temperature of 42/37°C, with relative humidity maintained at 60%–70%. The photoperiod was set to 16 hours of light and 8 hours of darkness, with a light intensity of 30 000 lux. The treatment lasted for 12 days, with leaf samples collected on days 0, 6, and 12 for the analysis of *PlSGRL* and *PlMYB3RL* expression.

### Total RNA extraction and reverse transcription quantitative PCR analysis

Total RNA from leaves, stems, and roots was extracted using a commercial RNA extraction kit (TaKaRa, Beijing, China). Subsequently, the extracted RNA was reverse transcribed into first-strand cDNA using a cDNA Synthesis SuperMix specifically designed for qPCR (TransGen, Beijing, China). Reverse transcription quantitative PCR (RT-qPCR) analyses were conducted on a CFX96 Touch™ Real-Time PCR Detection System (Bio-Rad, CA, USA) using PerfectStart® Green qPCR SuperMix (TransGen, Beijing, China). The internal reference gene, *PlActin* (GenBank accession JN105299), was employed, and the 2^-ΔΔCt^ method was used to quantify relative gene expression. The primers used in this study are listed in Supplementary Table S1.

### Gene and promoter cloning

The CDSs of *PlSGRL* and *PlMYB3RL* were cloned via PCR with primers designed according to the *P. lactiflora* transcriptome database (NCBI SRA: PRJNA1059272). Multiple sequence alignment of PlSGRL and PlMYB3RL homologs was conducted using ClustalW in MEGA 7.0. Based on this, the phylogenetic tree was constructed using the neighbor-joining method with 1000 bootstrap replicates. The promoter of *PlSGRL* was amplified using the chromosome walking method with the Genome Walker Universal Kit, utilizing DNA extracted from *P. lactiflora* as the template.

### Subcellular localization

The CDSs of *PlSGRL* and *PlMYB3RL* were cloned into the pCAMBIA2300-eGFP vector, and recombinant plasmids, 35S::PlSGRL-eGFP and 35S::PlMYB3RL-eGFP, along with the empty pCAMBIA2300-eGFP vector, were transformed into *Agrobacterium tumefaciens* strain GV3101. These strains were then co-infiltrated into 40-day-old *N. benthamiana* leaves alongside a nuclear-localized mCherry fusion (*A. thaliana* histone H2B, GenBank NM_122194.3). Fluorescence signals were detected two days post- infiltration using a confocal laser scanning microscope (Leica TCS SP8, Wetzlar, Germany). The excitation light wavelength for GFP was 488 nm, and the emission light wavelength ranged from 494 to 563 nm. For mCherry, the excitation light wavelength was 561 nm, and the emission light wavelength ranged from 567 to 635 nm. For chlorophyll autofluorescence, the excitation light wavelength was 633 nm, and the emission light wavelength ranged from 650 to 750 nm.

### Gene silencing in *P. lactiflora*

To silence *PlSGRL* and *PlMYB3RL*, target-specific fragments were cloned into the pTRV2 vector. Following our established protocol, *A. tumefaciens* strains harboring either the empty pTRV2 vector or the pTRV2-gene fragment plasmids were mixed with pTRV1-containing strains and co-infiltrated into the root systems of *P. lactiflora* [53]. After positive identification by PCR using specific primers for the inserted gene fragments and RT-qPCR to confirm the down-regulation of target genes, the transformed plants were exposed to high-temperature stress following the same protocol described earlier. Finally, phenotypic observations and measurements of related physiological parameters were performed.

### Gene overexpression in *N. tabacum*

The CDS of *PlSGRL* was inserted into the pCAMBIA1301 vector under the CaMV 35S promoter. Transgenic *N. tabacum* ‘K326’ plants were then generated using the leaf-disc transformation method described by Sunilkumar et al. [54] and confirmed by PCR detection. Following 1–2 months of growth, the positive transgenic plants were exposed to high-temperature stress. The treatment conditions and measured parameters were consistent with those used in the *P. lactiflora* experiment.

### Y1H assay

The 2218-bp *PlSGRL* promoter was inserted into the pAbAi vector, followed by transformation into the Y1HGold yeast strain. A *P. lactiflora* cDNA library cloned into pGADT7 was screened for interacting transcription factors. To identify the optimal concentration of AbA required to suppress self-activation of the bait vector, the Y1HGold yeast strains harboring the bait vector (designated as bait strains) were cultured on SD/-Ura medium amended with a range of AbA concentrations. Following this, the bait strains were transformed with the cDNA library and then incubated in SD/-Leu medium containing AbA at the ascertained optimal concentration. Positive clones were identified by sequencing and BLAST analysis, and *PlMYB3RL* was selected for further study. Finally, yeast bait strains harboring pGADT7-PlMYB3RL or empty pGADT7 (negative control) were cultured on SD/-Leu medium containing AbA at optimized concentrations to evaluate the interactions between proteins and promoters.

### DLR assay

The CDS of *PlMYB3RL* and *PlSGRL* promoter regions were cloned into the pGreenII 0062-SK effector and pGreenII 0800-LUC reporter vectors, respectively. *Agrobacterium tumefaciens* GV3101 strains were transformed with either recombinant effector constructs or empty pGreenII 0062-SK control. These strains were co-infiltrated into *N. benthamiana* leaves at a volume ratio of 10:1 (effector:reporter). At 48 hours post-infiltration, the relative activities of LUC and REN were measured with the detection reagents, followed by LUC signal imaging after the application of 1 mM D-luciferin potassium salt substrate.

### Y2H assay

The CDS of *PlMYB3RL* was cloned into the bait vector pGBKT7 and cotransformed with a *P. lactiflora* cDNA library prey vector into Y2HGold yeast strain. Positive interactors were selected on SD/-Trp-Leu-His-Ade medium supplemented with X-α-gal and 50 ng mL^−1^ AbA, and their identities were verified by sequencing. PlMYB3RL was chosen for further investigation. The *PlMYB3RL* CDS was subsequently cloned into the pGADT7 vector. Self-interaction was confirmed by cotransforming pGBKT7-PlMYB3RL (bait) and pGADT7-PlMYB3RL (prey) into Y2HGold yeast strains under the same selection conditions.

### BiFC assay

The CDS of *PlMYB3RL* was inserted into both the pSPYNE and pSPYCE vectors. *Agrobacterium tumefaciens* GV3101 strains were co-infiltrated into *N. benthamiana* leaves using the following combinations: pSPYNE-PlMYB3RL + pSPYCE-PlMYB3RL (experimental), pSPYNE-PlMYB3RL + empty pSPYCE (control), and empty pSPYNE + pSPYCE-PlMYB3RL (control). Following a 48-hour incubation, yellow fluorescent protein and mCherry fluorescence signals were detected using excitation wavelengths of 514 nm and 561 nm, with emission collected through 522–595 nm and 567–635 nm bandpass filters, respectively.

### LCA assay

The CDS of *PlMYB3RL* was cloned into the nLUC and cLUC vectors. *Agrobacterium tumefaciens* strains transformed with the recombinant plasmids or empty nLUC and cLUC vectors were co-infiltrated into *N. benthamiana* leaves. After 48 hours, LUC fluorescence was imaged following the application of 1 mM D-luciferin potassium salt substrate.

### Physiological index measurement

Leaf Fv/Fm values were determined using a PlantView 230F Chlorophyll fluorometer (BLT, Guangzhou, China). REC levels were measured with a DDS-307A conductivity meter (Leici, Shanghai, China). MDA content was quantified using commercial detection kits (Komin, Suzhou, China). O_2_^·-^ was detected by NBT staining, and H_2_O_2_ was visualized using DAB staining. Chlorophyll a and b contents were measured following our previously described method [35]. Leaf color parameters, including a* (red-green axis), b* (yellow-blue axis), and *h*^°^ (hue angle), were determined from the third fully expanded leaf using a colorimeter (X-Rite, Shanghai, China).

### Statistical analysis

All data were statistically analyzed by one-way analysis of variance with Tukey’s HSD *post-hoc* test (*p* < 0.05) using SPSS 25.0 software (IBM, Armonk, USA). At least three biological replicates were set for each treatment group. Graphs were generated using GraphPad, and error bars stand for the standard deviation.

## Supplementary Material

Web_Material_uhag135

## Data Availability

All relevant data can be found within the manuscript and its supporting materials.
