# Supplementary material for: PlMYB3RL homodimer activates PlSGRL to accelerate high temperature–induced leaf senescence by promoting chlorophyll degradation in herbaceous peony (Paeonia lactiflora Pall.)
Source: Hortic Res. 2026 Apr 13;13(8):uhag135. doi: 10.1093/hr/uhag135 (PMC13438600; doi:10.1093/hr/uhag135)
Supplement: Supplementary file 1 [file web_material_uhag135.zip › Supplementary Figure.docx]

**Figure S1**

**Figure S1.** PCR and RT-qPCR identification of the *PlSGRL*-silenced *P. lactiflora* plants based on VIGS. pTRV, empty vector. The values represent the means ± SDs, and different letters indicate significant differences (*P* < 0.05).

**Figure S2**

**Figure S2.** Changes in leaf color parameters of the *PlSGRL*-silenced *P. lactiflora* plants. *a**, red-green axis; *b**, yellow-blue axis; *h*^°^, hue angle. The values represent the means ± SDs, and different letters indicate significant differences (*P* < 0.05).

**Figure S3**

**Figure S3.** PCR Identification of the *PlSGRL* transgenic *N. tabacum* plants. Hyg, hygromycin; WT, wild-type.

**Figure S4**

**Figure S4.** Changes in leaf color parameters of the *PlSGRL*-overexpressing *N. tabacum* plants. *a**, red-green axis), *b**, yellow-blue axis, and *h*^°^, hue angle. The values represent the means ± SDs, and different letters indicate significant differences (*P* < 0.05).

**Figure S5**

**Figure S5.** Sequence alignment of PlMYB3RL and *Arabidopsis thaliana* At5g41020.

**Figure S6**

**Figure S6.** PCR and RT-qPCR identification of the *PlMYB3RL*-silenced *P. lactiflora* plants based on VIGS. pTRV, empty vector. The values represent the means ± SDs, and different letters indicate significant differences (*P* < 0.05).

**Figure S7**

**Figure S7.** Changes in leaf color parameters of the *PlMYB3RL*-silenced *P. lactiflora* plants. *a**, red-green axis; *b**, yellow-blue axis; *h*^°^, hue angle. The values represent the means ± SDs, and different letters indicate significant differences (*P* < 0.05).
